# Effect of HPSE and HPSE2 SNPs on the Risk of Developing Primary Paraskeletal Multiple Myeloma

**DOI:** 10.3390/cells12060913

**Published:** 2023-03-16

**Authors:** Olga Ostrovsky, Katia Beider, Hila Magen, Merav Leiba, Ralph D. Sanderson, Israel Vlodavsky, Arnon Nagler

**Affiliations:** 1 Department of Hematology and Bone Marrow Transplantation, Chaim Sheba Medical Center, Tel-Hashomer 5266202, Israel; 2 Department of Pathology, University of Alabama at Birmingham, Birmingham, AL 35294, USA; 3 Technion Integrated Cancer Center (TICC), Rappaport Faculty of Medicine, Technion, Haifa 3525433, Israel

**Keywords:** HPSE gene, HPSE2 gene, SNPs, multiple myeloma, extramedullary disease

## Abstract

Multiple myeloma (MM) is a plasma cell malignancy that is accompanied by hypercalcemia, renal failure, anemia, and lytic bone lesions. Heparanase (HPSE) plays an important role in supporting and promoting myeloma progression, maintenance of plasma cell stemness, and resistance to therapy. Previous studies identified functional single nucleotide polymorphisms (SNPs) located in the HPSE gene. In the present study, 5 functional HPSE SNPs and 11 novel HPSE2 SNPs were examined. A very significant association between two enhancer (rs4693608 and rs4693084), and two insulator (rs4364254 and rs4426765) HPSE SNPs and primary paraskeletal disease (PS) was observed. SNP rs657442, located in intron 9 of the HPSE2 gene, revealed a significant protective association with primary paraskeletal disease and lytic bone lesions. The present study demonstrates a promoting (HPSE gene) and protective (HPSE2 gene) role of gene regulatory elements in the development of paraskeletal disease and bone morbidity. The effect of signal discrepancy between myeloma cells and normal cells of the tumor microenvironment is proposed as a mechanism for the involvement of heparanase in primary PS. We suggest that an increase in heparanase-2 expression can lead to effective suppression of heparanase activity in multiple myeloma accompanied by extramedullary and osteolytic bone disease.

## 1. Introduction

Multiple myeloma (MM) is a malignant neoplasm of plasma cells. The disease is defined by the presence of 10% or more clonal plasma cells in the bone marrow (BM), accompanied by one or more myeloma-defining events (MDE) consisting of the CRAB criteria (hypercalcemia, renal failure, anemia, and lytic bone lesions), and three specific biomarkers: clonal bone marrow plasma cells ≥ 60%, serum free light chain (FLC) ratio ≥ 100, and more than one focal lesion in MRI [1,2]. A subclone of plasma cells, capable of growing outside the BM, may lead to extramedullary multiple myeloma (EMD) [3]. Extramedullary myeloma, presented at initial diagnosis (primary EMD) or recurrence (secondary EMD), includes two categories: i. Extramedullary disease (EMD), an aggressive form of MM with soft-tissue plasmacytomas resulting from hematogenous spread; ii. Paraskeletal disease (PS) characterized by the presence of soft-tissue plasmacytomas arising from direct growth of skeletal tumors with distraction of the bone cortex. Solitary plasmacytoma (SP) and plasma cell leukemia (PCL) are typically excluded from the definition of EMD [3,4]. Despite the implementation of new therapeutic agents, MM is still an incurable disease due to recurrent relapses in most patients [5].

Heparanase, the sole heparan sulfate (HS) degrading endoglycosidase, plays an important role in supporting and promoting myeloma progression, maintenance of cancer cell stemness, and resistance to chemotherapy [6,7,8]. High heparanase expression enhances bone marrow angiogenesis, MM growth and osteolytic bone disease [9,10,11]. Heparanase leads to structural modifications of syndecan-1 resulting in syndecan-1 shedding from the myeloma cell surface [12,13,14]. High levels of shed syndecan-1 are correlated with poor prognosis and overall survival of MM patients [12,13,14]. Moreover, heparanase-dependent activation of the ERK and p38 signaling pathways is associated with myeloma progression [13,15]. In addition, heparanase is an important factor in the biogenesis and functioning of exosomes, which influence the control of cell behavior in the myeloma microenvironment and distal sites [16,17]. Nuclear heparanase enhances chromatin remodeling, stimulates gene transcription involved in MM progression, and blocks the tumor suppressor function of PTEN [18]. 

The HPSE2 gene encodes a close heparanase homolog, named heparanase-2 (Hpa2) [19]. Unlike the intense research effort devoted to exploring the significance of heparanase (Hpa1) in human diseases, very little attention was given to heparanase-2 (Hpa2). Hpa2 gained attention when it was reported that the HPSE2 gene is mutated in a human disease called urofacial syndrome (UFS) [20,21]. UFS is a rare autosomal recessive congenital disease, featuring a combination of urological defects and an inverted facial expression attributed to peripheral neuropathy [22]. Hpa2 lacks intrinsic heparan sulfate (HS)-degrading activity, binds heparin/HS with high affinity, and thereby competes for HS binding and inhibits heparanase enzymatic activity [23]. Co-immunoprecipitation revealed a physical association between the two proteins [23]. The role of Hpa2 in the development of cancer is not well understood. However, heparanase-2 inhibits heparanase enzymatic activity [23] and appears to function as a tumor suppressor [19,24,25,26,27,28,29]. The role of heparanase-2 in multiple myeloma has not been elucidated. 

Previously, several functional single nucleotide polymorphisms (SNPs) located in the enhancer and insulator of the HPSE gene have been identified [30,31], the most prominent of which are rs4693608 (enhancer) and rs4364254 (insulator) SNPs [30,31]. Combinations of rs4693608 and rs4364254 SNPs are significantly associated with high (HR), intermediate (MR) and low (LR) HPSE gene levels in normal leukocytes and mononuclear cells (MNCs) in both peripheral and umbilical cord blood [32,33]. The HR group includes all individuals with the rs4693608 AA genotype. The MR group consists of carriers AG-TT and AG-TC genotypes, and the LR group includes the genotypes GG-TT, GG-TC, GG-CC, and AG-CC. Moreover, a highly significant association was found between rs4693608 and the risk of acute and extensive chronic graft versus host disease (GVHD) after allogeneic stem cell transplantation (HSCT). Importantly, the discrepancy between the recipient and donor in these SNPs significantly affects the risk of acute GVHD [33,34]. Notably, the enhancer rs4693084 SNP and insulator rs28649799 SNP were shown to be associated with the yield of G-CSF-mediated CD34^+^ cell mobilization in normal donors [31,35]. In addition, rs4426765 is associated with heparanase gene expression in activated MNCs, as well as CD3 cell number and lymphocyte count in response to G-CSF-induced cell mobilization [31]. 

In the present study, the functional SNPs of the HPSE and HPSE2 genes were analyzed. For the first time, 11 SNPs of HPSE2 were examined. Our results show a very significant association between two enhancer (rs4693608 and rs4693084), and two insulator (rs4364254 and rs4426765) HPSE SNPs and primary paraskeletal disease (PS). SNPs rs4693608 and rs4364254 exhibited the most significant results. The frequencies of the LR genotype, previously shown to be associated with a low level of HPSE expression and a low risk of developing acute GVHD [32,33,34], were significantly higher in the group with total and paraskeletal disease compared with control patients. Significant correlations were found with rs4693608, rs4693084, and rs4426765 SNPs and multiple myeloma stage at diagnosis. SNP rs657442, located in intron 9 of the HPSE2 gene, revealed a significant protective association with primary PS disease and lytic bone lesions. The impact of rs658225 and rs754585 HPSE2 gene SNPs was noted in secondary extramedullary disease.

The present study demonstrates the promoting (HPSE) and protective (HPSE2) role of regulatory gene elements in the development of paraskeletal disease and bone morbidity. We suggest that signal discrepancy between malignant myeloma cells and normal cells of the microenvironment provides a possible mechanism for the involvement of heparanase in primary EMD.

## 2. Materials and Methods

### 2.1. Study Population

Two hundred and seventy eight patients were included in the study. Nineteen patients were diagnosed with monoclonal gammopathy (MGUS) and 28 with smoldering multiple myeloma (SMM). Six patients with MGUS and 4 with SMM progressed to active multiple myeloma (MM). Clinical and disease characteristics, including disease stage by the International Staging System (ISS), bone involvement, renal failure, extramedullary disease, beta-2-microglobulin, creatinine, albumin, calcium, and hemoglobin levels were collected from medical records. The definition of renal failure is based on an estimated glomerular filtration rate (eGFR) or creatinine clearance (CrCl) of 29–15 mL/min/1.73 m^2^. Patient characteristics are presented in Table 1. The study was approved by the Sheba Medical Center Ethics Committee and the Israeli Ministry of Health (#4247) and all subjects gave their written informed consent.

### 2.2. SNPs Analysis

Genomic DNA was extracted from peripheral blood applying the Wizard^®^ Genomic DNA Purification kit (Promega, Madison, WI, USA). Functional SNPs of the HPSE and HPSE2 genes were genotyped using Real-Time SNP Assay (Bio Search Technologies, Novato, CA, USA) and TaqMan SNP Genotyping Assay (Thermo Fisher Scientific, Pleasanton, CA, USA). PCR reactions were performed using an ABI PRISM 7700 sequence detector (Applied Biosystems, Warrington, UK) according to the manufacturer’s instructions (Quanta, Gaithersburg, MD, USA).

### 2.3. DNA Constructs

A fragment of the HPSE gene enhancer (440 bp), located in intron 2 and including rs4693083, rs4693608, rs4693084 and rs4693609 SNPs, was cloned via PCR using PCR II-TOPO vector (Invitrogen by Life Technologies, Carlsbad, CA), as previously described in detail [30]. The multiple myeloma cell line RPMI-8226, isolated from a patient with plasmacytoma, was seeded into six-well culture dishes according to Lonza instructions (AMAXA biosystems, Lonza, Germany). Cells were transfected with 2 µg of each DNA construct using the Ingenio Electroporation Kit (Mirus Bio, Madison, WI, USA) and Nucleofector^TM^ (AMAXA biosystems, Lonza, Germany). Twenty-four hours after electroporation, the cells proceeded to RNA analysis. 

### 2.4. Purification of Total RNA, Generation of cDNA, and Real-Time Quantitative RT-PCR

Total RNA was obtained from RPMI-8226 cell line using the TRIzol reagent (Invitrogen, Carlsbad, CA, USA) according to the manufacturer’s instructions. Complementary DNA (cDNA) was synthesized using 1µg total RNA applying the qScript cDNA Synthesis kit (Quanta, Gaithersburg, MD, USA). PerfeCTa SYBR Green Fast Mix ROX (Quanta, Gaithersburg, MD, USA) Real-time PCR reactions to amplify the HPSE and HPSE2 genes and the RPL-19 housekeeping gene were performed using an ABI PRISM 7700 sequence detector (Applied Biosystems, Warrington, UK) in a total volume of 20 µL. The primer sequences for HPSE and RPL-19 genes were previously published [36]. HPSE2 expression was determined using primers 5’CAGGGCATTGATGTCGTGATAC3’ and 5’GCCAGTAGTCTGGTAATGGGTTA3’. The experiments were performed in triplicates and a standard curve was created. A dissociation curve was generated after each experiment to confirm that one product was amplified. Comparative CT method (∆∆CT) was used to quantify HPSE and HPSE2 gene expression, and relative quantification (RQ) was calculated as 2^−∆∆CT^.

### 2.5. Statistical Analysis

All calculations were performed using the NCSS software (NCSS, Kaysville, Utah, USA). A *p*-value ≤ 0.05 was considered statistically significant. Genotype and allele frequencies of the SNPs were calculated by direct counting. Possible differences between samples in the frequency of each SNP genotype or allele were assessed using the χ^2^ test. The Yates correction was used when analyzing the interaction of two or more SNPs. Due to the low frequency of allele A of SNP rs657422, association analysis was carried out using Fisher’s exact test.

## 3. Results

### 3.1. Analysis of HPSE and HPSE2 Gene SNPs among Multiple Myeloma Patients

**Figure 1:**
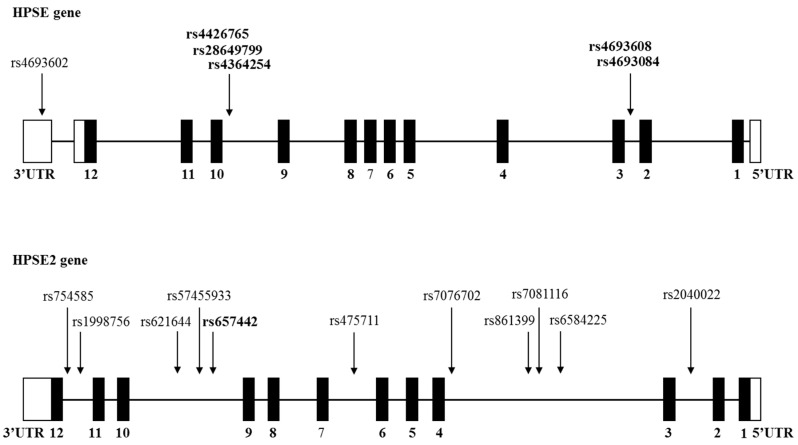
**Map of the HPSE and HPSE2 genes.** Numbered boxes indicate exons. Black boxes depict open reading frames, and white boxes represent the 5’- and 3’-untranslated regions. The most prominent SNPs are marked in bold in both genes. Arrows point to the location of SNPs.

The retrospective study included 278 multiple myeloma patients (174 males and 104 females) and 211 controls. Two hundred and thirty one patients were diagnosed with multiple myeloma, 28 with SMM and 19 with MGUS. The median age was 60 years (range 27–90). All statistical analyzes, except for association with the patient’s diagnosis (MM, SMM and MGUS), were performed in 259 patients who developed active MM. Patient characteristics are presented in Table 1. Six HPSE gene SNPs were used in the case vs control analyses. Two functional SNPs rs4693608 and rs4693084 are located in the HPSE gene enhancer, three functional SNPs (rs4426765, rs28649799 and rs4364254) are mapped in the HPSE gene insulator, and rs4693602 is situated in the 3’UTR (Figure 1). SNPs of the HPSE2 gene were analyzed for the first time. Eleven SNPs that could potentially be functional polymorphisms or be associated with functional SNPs were selected (Figure 1). A correlation analysis was performed between the HPSE and HPSE2 SNPs and the patient’s diagnosis (MM, SMM, and MGUS). The frequency of the TT genotype (rs4364254) was slightly higher in patients with MM compared to the control group of healthy individuals, but statistical analysis showed only a trend toward correlation (Supplementary Table S1). No associations were observed for rs4364254 allele frequencies (Supplementary Table S1). In addition, no correlations were found between the HPSE2 gene SNPs and the patient’s diagnosis.

### 3.2. Association between HPSE Gene SNPs and Stage of Multiple Myeloma Patients at Diagnosis

The influence of HPSE and HPSE2 SNPs on the stage of multiple myeloma at diagnosis was analyzed in 151 patients. The stage was determined according to the ISS [37]. Stage I was diagnosed in 25% of patients, stage II in 43.7%, and stage III in 30.5%. The association between HPSE gene SNPs and MM staging is shown in Table 2. Significant correlations were found with enhancer rs4693608 and rs4693084 SNPs and insulator rs4426765 SNP. The most significant results were obtained in comparing stage I and stage II/ III. The frequencies of the AA genotype were higher in patients diagnosed with stage II and III (15.4% in stage I vs. 40.9% and 32.6% in stages II and III, respectively). The frequencies of the GG genotype were higher in patients diagnosed with stage I vs II and III (25.6% at stage I vs. 12% and 15.2% at stages II and III, respectively). The *p*-value for genotype frequency was *p* = 0.022 and the *p*-value for allele frequency was *p* = 0.0082 (Table 2). No correlations with HPSE2 gene SNPs were found.

### 3.3. Association between HPSE Gene SNPs and Primary Bone-Related Disease in Multiple Myeloma

Fifty patients with primary extramedullary disease were identified. Forty-one of 50 were diagnosed with paraskeletal disease, one with EMD from hematogenous spread and two had both bone-depended and bone-independent EMD. One patient was diagnosed with solitary plasmacytoma, and five developed plasma cell leukemia. 

Analysis of the correlation between functional SNPs of the HPSE gene is presented in Table 3. Calculations were performed for all individuals diagnosed with any type of EMD and separately for patients who developed paraskeletal disease. Four functional SNPs revealed highly significant associations (Table 3). The frequency of the GG genotype and the G allele of rs4693608 SNP was higher in patients with EMD compared to the control group (32% versus 13% for the GG genotype, 18% versus 36.4% for the AA genotype, *p* = 0.003, and 57% versus 38.3%, *p* = 0.001 for the G allele, respectively, Table 3). Similar results were obtained for paraskeletal disease (*p* = 0.018 for genotype comparison and *p* = 0.006 for allele comparison) (Table 3). All three insulator SNPs were associated with EMD, rs4364254 being the most prominent SNP (Table 3). The frequency of the CC genotype and the C allele of rs4364254 SNP was higher in patients with primary total EMD and patients with paraskeletal disease (20% versus 8.6%, *p* = 0.003 for the CC genotype; 43% versus 24.4%, *p* = 0.00032 for allele C in total EMD; 18.6% versus 8.6%, *p* = 0.019 for genotype CC; and 40.7% versus 24.4%, *p* = 0.003 for allele C in paraskeletal disease, respectively, Table 3). 

In our previous studies, we investigated the interaction between enhancer and insulator HPSE gene SNPs [31,32,34]. We allocated all possible HPSE genotype combinations into three groups (HR, MR, and LR) correlating with high, intermediate and low heparanase mRNA expression levels and with high, intermediate and low risk of acute GVHD, respectively [31,32,34]. Given the results presented in Table 3, we can conclude that rs4693608 and rs4364254 exhibited the most significant association with EMD. Therefore, correlations were analyzed in HR (genotypes AA-TT and AA-TC), MR (genotypes AG-TT and AG-TC), and LR (genotypes GG-TT, GG-TC, GG-CC and AG-CC) groups. Frequency of the LR genotype was significantly higher in patients with total and paraskeletal disease compared with patients without EMD disease (44% for total EMD and 41.9% for paraskeletal disease versus 16.1% in the other patients, χ^2^ = 16.036, *p* = 0.00033 for total EMD and χ^2^ = 14.32, *p* = 0.002 for paraskeletal disease after Yates’ correction) (Table 3).

### 3.4. Association between HPSE2 Gene SNPs and Primary Extramedullary Disease (EMD) in Multiple Myeloma

Eleven HPSE2 SNPs were analyzed. Only one SNP, rs657442, located in intron 9, demonstrated a significant correlation with primary EMD (Table 4). Of note, all patients (100%) diagnosed with EMD were carriers of the CC genotype, while in the control group, only 86.4% were carriers of this genotype (*p* = 0.0065 for total EMD and *p* = 0.011 for paraskeletal disease). The A allele frequency was 7.1% in the control group and null in the EMD group (*p* = 0.038 for total EMD and *p* = 0.0066 for paraskeletal disease, Table 4). 

The interactions between HPSE genotypes and the significant HPSE2 SNP rs657442 are presented in Table 5. A protective effect of the A allele was observed. Five patients with the LR genotype, identified in association with the risk of EMD, were carriers of the A allele for SNP rs657442 and had neither primary nor secondary EMD (Table 5). Primary EMD was not observed in 8 carriers of the A allele and MR genotype. Three of them developed secondary EMD, one with hematogenous spread and two with paraskeletal disease. The frequency of the A allele is low, and there are many variations with null frequencies (Table 5). Therefore, the impact of HPSE-HPSE2 gene interaction was assessed by allocation of all possible variants into three groups with high-, intermediate- and low-risk. In group A, we hypothesized that the protective effect of the A allele could downgrade the risk of developing EMD from high to intermediate, and from intermediate to low. Therefore, group A1 included the HR-CC, HR-CA, HR-AA, and MR-CA genotypes. The LR-CA variant was added to group A2. Since we could not assess the magnitude of the A allele-mediated protective effect against EMD in group B, we assumed that the protective effect was high in all subjects with the A allele. As a result, the LR-CA and MR-CA genotypes were included in group B1. Chi-square analysis was performed for both groups, and after Yates’ correction, the summarized effect was found to be highly significant compared to the *p*-values for each SNP alone (*p* = 0.000015 for group A and *p* = 0.0000098 for group B) (Table 5).

### 3.5. Association between HPSE2 Gene SNPs and Secondary Extramedullary Disease (EMD) in Multiple Myeloma

Thirty-eight patients developed secondary EMD. Seven of them had soft-tissue plasmacytomas with hematogenous spread, 27 had bone-related EMD, and 4 had both types of EMD. No correlation with HPSE gene SNPs was observed. Analysis of the association between HPSE2 gene SNPs and secondary EMD is shown in Table 6. Some associations were identified with rs658225 (intron 3), rs621644 (intron 9), and rs754585 (intron 11) (Figure 1 and Table 6). Notably, the G allele of rs658225 SNP exhibited a protective effect. The G allele frequency in the control group (18%) was higher compared to patients with secondary EMD (8.1%) (*p* = 0.03, Table 6). In addition, the TT genotype of rs754585 SNP disclosed a protective effect (33.3% in the control group vs. 13.5% in the secondary EMD group, *p* = 0.027). However, the frequency of the CC genotype was the same in both groups (Table 6). An opposite effect was observed in heterozygous individuals (64.9% in the secondary EMD group vs. 41.7% in the control group). 

The interaction between these SNPs was assessed (Table 7). Since the number of patients with secondary EMD was relatively small, the analysis was carried out not only in the group of secondary EMD but also for all patients who revealed EMD. The same trend was observed both in total and secondary EMD compared with the control group. The protective effect of the G allele was manifested in the possessors of the AG genotype for rs658225 (Table 7).

The influence of SNP rs754585 was demonstrated in carriers of the AA genotype. The risk of secondary EMD in patients with the AA-TT genotype was low compared with the control group (10.5% vs. 22.5%), while in individuals with the AA-CT genotype the risk of secondary EMD was higher (52.6% in the secondary EMD group vs. 23.3% in the control group). No differences were found among AA-CC carriers. Due to the small number of individuals with the AG-TT genotype, it was impossible to confirm an addictive apologetic effect of rs658225 and rs754585 HPSE2 SNPs (Table 7). Additional studies are needed in a larger cohort of patients with EMD. 

### 3.6. Association between HPSE2 Gene SNPs and Bone Morbidity in Multiple Myeloma Patients

One hundred and fifty-eight multiple myeloma patients (74.9%) exhibited one or more osteolytic bone lesions, while 53 patients (25.1%) had no lytic bone disease. Analysis of the functional HPSE gene SNPs did not reveal a correlation with bone involvement. Eleven SNPs of the HPSE2 gene were genotyped among patients with multiple myeloma. A highly significant correlation with bone morbidity was observed with rs657442 SNP (intron 9), a SNP with a relatively rare frequency of the A allele. Only one individual was genotyped as homozygous for the A allele. Therefore, the following comparison is related to the frequencies of the CA genotype and the A allele. Only 5.7% of patients with the CA genotype had bone involvement compared to 20.8% of patients who had no detectable bone disease (*p* = 0.001 for genotype frequency, and *p* = 0.00016 for allele frequency, Table 8). Collectively, HPSE2 gene SNP rs657442 revealed a protective effect.

### 3.7. Modification of HPSE and HPSE2 Gene Expression after Transient Transfection of RPMI-8226 MM Cells with a Fragment of the Enhancer

**Figure 2:**
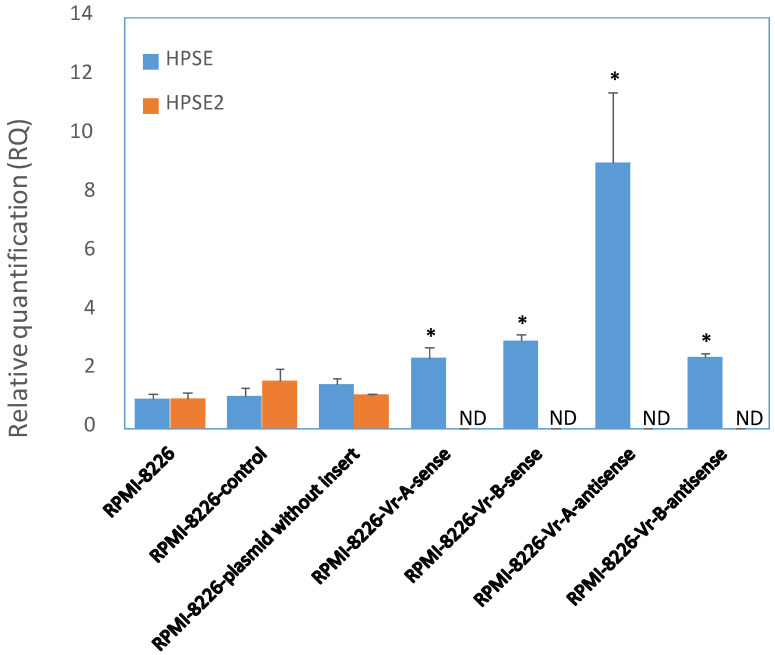
Expression of the HPSE and HPSE2 genes in multiple myeloma RPMI-8226 cells transfected with the HPSE gene enhancer fragment. Four HPSE enhancer constructs were inserted upstream of the luciferase gene in pGL4.26 vector with minimal promoter and transfected into RPMI-8226 myeloma cells [30]. DNA constructs were prepared in sense and antisense direction. The HPSE gene enhancer fragment (440 bp) included rs4693083, rs4693608, rs4693084, and rs4693609 SNPs. Vr-A construct contains allele A of rs4693608 and allele G of rs4693084. Vr-B fragment includes allele G of rs4693608 and allele T of rs4693084. Samples of RPMI-8226 cells before and after transfection were analyzed by quantitative RT-PCR. RQ (relative quantification) values are shown on the y-axis. HPSE gene expression levels were elevated in enhancer fragment-transfected samples compared to controls, while HPSE2 gene expression was decreased to undetectable levels in the same samples. Samples treated with AMAXA electrical pulse alone or transfected with empty plasmid were used as controls. No differences were observed between untreated and treated samples. Data shown are of representative experiments performed in triplicates. Differences in RQ among various constructs were evaluated by *t*-test. *p* value of ≤0.05 was regarded as statistically significant. * *p* value of ≤0.05. ND—not detected.

In a previously published study, we found an active HPSE enhancer in intron 2 of the HPSE gene [30]. To determine the functional effects of enhancer SNPs, we applied a luciferase reporter gene with a minimal promoter to measure enhancer activity. Briefly, RPMI-8226 cells were transiently transfected with DNA constructs in the sense and antisense directions or with an empty vector [30]. As an additional control, RPMI-8226 cells were subjected to an electric pulse. The HPSE gene enhancer fragment (440 bp) included rs4693083, rs4693608, rs4693084, and rs4693609 SNPs. We have previously reported [30] that the 440 bp fragment of the HPSE gene exhibits enhancer activity in both the sense and antisense directions. In the present study, we evaluated the effect of the enhancer fragment transfection on the expression of HPSE and HPSE2 and the results are presented in Figure 2. HPSE expression was increased in all the transfected samples, while the level of the HPSE gene remained unchanged in the control samples.

The expression level of the HPSE2 gene is low in most malignant cell lines [19,20,21,22,23,24,25,26,27,28,29]. Likewise, RMPI-8226 cells expressed a very low, but detectable level of HPSE2 and there was no effect to electrical pulse and transfection with an empty plasmid. Transfection of the HPSE enhancer fragment resulted in an undetectable level of HPSE2 expression (Figure 2), suggesting that the HPSE gene enhancer not only regulates HPSE gene expression but also has an opposite effect on the expression of the HPSE2 gene. The undetectable level of the HPSE2 gene makes it impossible to evaluate the role of specific HPSE enhancer functional SNPs in co-regulation of both genes.

## 4. Discussion

Association studies open up great prospects for elucidating the genetic basis of diseases. Moreover, functional SNP-based studies are effective because they focus on SNPs with the highest prior probability of being associated [38]. Functional SNPs located in regulatory elements, such as enhancers and insulators, help not only to indicate the involvement of a particular gene in the development of common diseases but also to explore the possible mechanisms of these enhancers or insulators in pathological processes [39,40]. Moreover, these regions can be used as targets for subsequent treatments [41].

In the present study, five previously identified and characterized functional HPSE gene SNPs were analyzed among patients with multiple myeloma. A highly significant association was found between the low-frequency alleles of rs4693608, rs4693084, rs4426765 and rs4364254 SNPs and an increased risk of primary paraskeletal disease. A previously published approach to studying the interaction between HPSE enhancer and insulator SNPs was applied [31,32,34]. The LR genotype, which correlates with low expression of the HPSE gene, was found to be associated with primary EMD.

Heparanase-2 appears to function as a natural inhibitor of the heparanase enzyme and a tumor suppressor [19,24,42]. While heparanase enhances the pathogenesis of cancer, heparanase-2 plays a protective role. It appears that the heparanase/Hpa2 ratio impacts tissue hemostasis [43,44] and the balance between cancer progression and suppression [19,24,42]. 

The present study aimed to identify and characterize possible functional SNPs located in the HPSE2 gene. Importantly, the rs657442 SNP, genotyped at intron 9, showed a protective effect on primary paraskeletal disease and bone-related morbidity. According to the ENCODE Registry of candidate cis-Regulatory Elements (cCREs), rs657442 SNP is located in a region with a distal enhancer-like signature (https://genome.ucsc.edu/cgi-bin/hgTracks?db=hg38&lastVirtModeType=default&lastVirtModeExtraState=&virtModeType=default&virtMode=0&nonVirtPosition=&position=chr10%3A98567418%2D98567918&hgsid=1502432943_OAIfBAS927s66nGeaamrwOHsFwZi, accessed on 20 May 2020). Additional three HPSE2 SNPs (rs658225, rs621644 and rs754585) revealed some correlation with secondary EMD. The rs658225 polymorphism was mapped to intron 3 and, according to the ENCODE Registry, is localized to the CTCF-related insulator (https://genome.ucsc.edu/cgi-bin/hgTracks?db=hg38&lastVirtModeType=default&lastVirtModeExtraState=&virtModeType=default&virtMode=0&nonVirtPosition=&position=chr18%3A43420931%2D43421131&hgsid=1502451125_iiE7HfeK7puYOrj2ozO3noTl2oPi, accessed on 20 May 2020). Intron 3 of the HPSE2 gene is a large intron that contains two pseudogenes and a gene encoding for MIR6507. It can be assumed that SNPs located in intron 3 may play an important role in the regulation of HPSE2 and other processes associated with MIR6507, and the RPL7P36 and ARL5AP2 pseudogenes. The rs624644 SNP has also been mapped to a region with a distal enhancer-like signature and could potentially be functional. SNP rs754585 was found to be in complete linkage disequilibrium with many polymorphic SNPs that are located in the 3’UTR region that can influence the expression and function of the HPSE2 gene. 

Heparanase is one of the main regulators of aggressive tumor behavior [7]. It drives angiogenesis, tumor growth, and metastasis [9,10,14,45,46,47,48]. Soluble heparanase is detected in the bone marrow of patients with myeloma associated with a high microvessel density [9]. Our results indicate that patients with genotypes AA-AA and AG-AC (rs4693608-rs4426765) had an increased risk of being diagnosed with stage II and III of the disease. Our previous studies revealed that the A allele is associated with higher heparanase levels in healthy individuals [32,33] and a higher risk of developing GVHD [34]. Andersen et al. identified a significant correlation between the A allele of rs4693608 and the risk of vertebral fractures at diagnosis [49]. Taken together, it is conceivable that the HPSE gene enhancer plays an important role in progression of the disease before diagnosis, and that the risk of multiple myeloma being diagnosed at later stages of the disease is higher in individuals with the AA genotype.

The interaction between myeloma cells and the bone marrow microenvironment promotes signaling cascades and activates chemotaxis and adhesion of myeloma cells to the bone marrow [50]. MM progression and extramedullary dissemination require continuous MM cell egress and spread into new extramedullary sites, regulated by dynamic changes in the expression of chemokine receptors and adhesion molecules. Thus, increased CXCR4/CXCL12 chemokine signaling in MM cells promotes epithelial to mesenchymal transition (EMT)-like phenotype with enhanced invasive properties [50,51,52,53,54]. Consequently, the CXCR4-mediated EMT-like program involves E-cadherin downregulation, resulting in decreased adhesion and enhanced egression of MM cells [54]. Furthermore, down-regulation of adhesion molecules (VLA-4, *p*-selectin, CD56, CD44), and membrane-embedded tetraspanins as well as up-regulation of heparanase are part of the mechanism regulating extramedullary spread in MM [3,18,53]. 

Heparanase is a multitasking protein whose activation promotes, among other effects, the release of various cytokines, chemokines and growth factors in the tumor microenvironment [9,55,56]. Moreover, nuclear heparanase in activated human T-lymphocytes modifies the activity of inducible immune response genes by association with euchromatin and regulation of histone H3 methylation [57]. ChIP-on-chip data revealed that heparanase bound to regulatory regions of myeloma-related genes (i.e., CXCL12) [57], encodes for SDF-1, CXCR4, CXCL2, CXCR3, CCL27, CX3CL1, and LCN2 [58,59,60]. The HPSE2 gene is also on this list [57], indicating that the interaction between HPSE and HPSE2 may occur at both the gene and protein levels [23]. This was confirmed our results. Transfection of HPSE gene enhancer to myeloma cells resulted in increased expression of the HPSE gene and subsequent downregulation of the HPSE 2 gene.

Unexpectedly, the present results indicate that MM patients with the LR genotype disclosed an increased risk of developing primary paraskeletal disease. Studies performed over the past two decades have shown that heparanase plays a major role in modulating the bone marrow microenvironment to support the progression of multiple myeloma [6]. High heparanase activity in myeloma cells correlates with myeloma growth, bone marrow angiogenesis, and osteolytic bone disease [6,9,11,12]. Our previous results showed that transfection of the HPSE enhancer and insulator fragments into RPMI-8226 and CAG human myeloma cells led to the highest level of luciferase activity compared to other similarly transfected cell types [30,31]. Thus, one would expect that the risk of developing a more aggressive disease in patients with HR genotype would be higher in comparison to carriers of the LR genotype. Indeed, a significant correlation was found between the rs4693608 AA genotype, which constitutes the bulk of the HR genotype, and a more progressive disease (stages II and III) at the time of diagnosis. 

**Figure 3:**
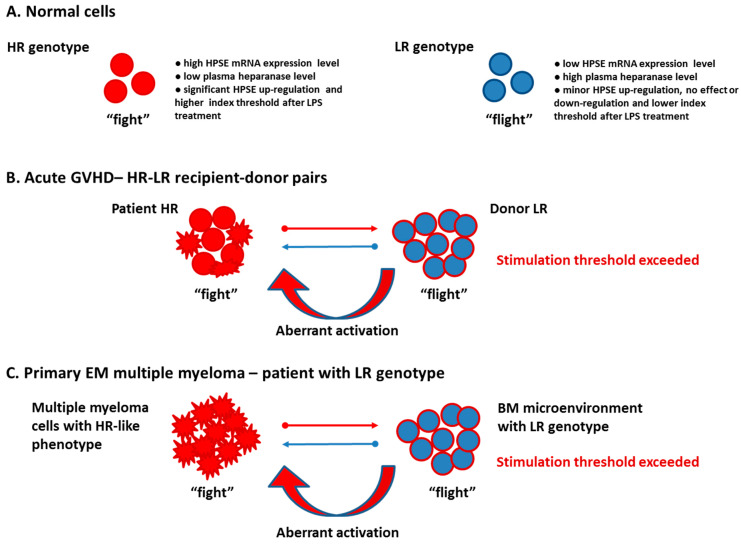
Scheme describing the putative role of discrepancy in HPSE SNP profiles as possible mechanisms for the development of acute GVHD and primary paraskeletal disease in multiple myeloma. (**A**). Effect of enhancer (rs4693608) and insulator (rs4364254) HPSE gene SNP combinations on heparanase expression in normal cells. (**B**). Proposed influence of HPSE gene SNP discrepancy between patient and donor on the development of acute GVHD following HSCT. (**C**). Discrepancy between myeloma and microenvironment signaling dictates an increased risk of developing paraskeletal disease in patients with the HPSE LR genotype (rs4693608-rs4364254 SNP combination).

To explain how the LR genotype increases the risk of paraskeletal disease, we propose the following mechanism, which is summarized in Figure 3. In our previous published studies [33,34], we found that the effect of a discrepancy between recipient and donor in HPSE gene SNPs leads to an increased risk of developing acute GVHD. The same effect was now observed in primary paraskeletal disease. To simplify, we adopt the concept of the “fight-or-flight” response to stress, first described by Walter Cannon [61]. Animals respond to stress with a set of predictable reactions known as “fight-or-flight” [62]. The function of the autonomic nervous system (ANS) in mediating the “fight-or-flight” response is well studied [63]. Briefly, the ANS is a major modulator of immune response and immune effector cells by activation of pro- and anti-inflammatory functions [64]. In the hematopoietic system, the ANS regulates stem cell niche homeostasis, and fine-tunes the inflammatory response [64]. Based on this approach, we named the HR genotype “fight” and the LR genotype “flight”. Our previous studies revealed that the HR genotype exhibited high HPSE mRNA expression, low plasma heparanase level, significant HPSE up-regulation, and most importantly, a higher index threshold after LPS treatment [32,33]. In contrast, the LR genotype disclosed low HPSE gene expression level, high plasma heparanase level, minor HPSE up-regulation, and no effect or downregulation of HPSE in response to LPS treatment [32,33]. A lower index threshold was observed (Figure 3A). Downstream heparanase signaling affects a variety of molecular mechanisms including transcription of immunologically related genes and miRNAs, and release of various cytokines, chemokines, and growth factors from the extracellular matrix [9,10,11,57]. Moreover, heparanase is an important modulator of exosome biogenesis via the syndecan-syntenin-ALIX pathway, affecting the level of exosomal cargo and the regulation of neurotransmission [65].

We suggest that the regulatory elements of the HPSE gene are involved in cell responsiveness to activating signals. After HSCT, engrafted donor cells (LR genotype), are exposed to the patient cells (HR genotype) and pro-inflammatory environment. When the stimulation threshold is exceeded, such donor cells with the LR “flight” genotype react with recipient cells harboring the HR “fight” genotype, resulting in aberrant activation and increased risk of developing acute GVHD increased (Figure 3B) [33,34].

We explain the identified risk of developing primary paraskeletal disease in patients with the LR genotype using a previously published discrepancy model [33,34]. Although aggressive myeloma cells exhibited the LR genotype, these cells have acquired a “fight” phenotype with high heparanase activity. In other words, while myeloma cells express HR-like phenotype, the normal microenvironment continues to express the LR genotype, leading to differences in signaling responses. When the signaling stimulation threshold is exceeded, aberrant activation of normal cells in the microenvironment results in bone cortex rupture and paraskeletal disease progression (Figure 3C). Previous studies have shown that heparanase expression by myeloma cells significantly increases local and systemic osteolysis [11]. In addition, heparanase can also alter the fate of osteoblast progenitor cells, directing cellular differentiation toward adipocytes rather than osteoblasts [66]. The effect of discrepancy in signaling between myeloma cells and the normal microenvironment, and the aberrant activity of cells in the bone marrow niche contribute to osteolysis, bone cortex distraction and various manifestation of bone-associated extramedullary disease.

The present study indicates the mutual role of HPSE and HPSE2 regulatory elements in the manifestation of multiple myeloma and proposes the use of heparanase 2-mimicking drugs for a most pronounced suppression of heparanase activity in multiple myeloma accompanied by EMD and osteolytic bone disease. 

## Figures and Tables

**Table 1:** Patient’s characteristics.

Variable	Characteristic	No. of Cases (%)
	median—60	
Age (years)	range—27–90	
	<60	128 (46.0)
	≥60	150 (54.0)
Gender	male	174 (62.6)
	female	104 (37.4)
MM	multiple myeloma	231(83.1)
SMM	smoldering multiple myeloma	28 (10.1)
MGUS	monoclonal gammopathy	19 (6.8)
ISS (international staging system)	I	39 (14.0)
	II	66 (23.7)
	III	46 (16.5)
	NA	127 (45.7)
Beta-2-microglobulin (µg/dL)	median—4.1	range—1.2–14.8
Creatinine (mg/dL)	median—1.04	range—0.5–20.0
Albumin (g/dL)	median—3.9	range—1.9–5.2
Calcium (mg/dL)	median—9.4	range—7.4–16.2
Hemoglobin (g/dL)	median—11.4	range—5.2–16.5
Bone morbidity	primary	149 (69.0)
	secondary	13 (6.0)
	without bone disease	54 (25.0)
Extramedullary disease	primary	50 (23.1)
	secondary	39 (18.1)
	without EMD	127 (58.8)
Renal failure	with renal failure	57 (26.3)
	without renal failure	160 (73.7)

ISS—international staging system; NA—data not available.

**Table 2:** Genotype and allele frequencies of the HPSE gene SNPs according to the International Staging System (ISS) in multiple myeloma patients.

SNPs/ Genotypes and Alleles	Stage I	Stage II	Stage III	χ^2^/ *p*-Value
	Number/ Incidence	Number/ Incidence	Number/ Incidence	
rs4693608 AA AG GG A G	6 (15.4%) 23 (59.0%) 10 (25.6%) 35 (44.9%) 43 (55.1%)	27 (40.9%) 31 (47.0%) 8 (12.0%) 85 (64.4%) 47 (35.6%)	15 (32.6%) 24 (52.2%) 7 (15.2%) 54 (58.7%) 38 (41.3%)	I − III: 3.86, 0.15 **I − II + III: 7.62, 0.022** I + II − III: 0.089, 0.96 I − III: 3.23, 0.072 **I − II + III: 6.99, 0.0082** I + II − III: 0.063, 0.8
rs4693084 GG GT TT G T	17 (43.6%) 18 (46.2%) 4 (10.3 %) 52 (66.7%) 26 (33.3%)	44 (66.7%) 19 (28.8%) 3 (4.5%) 107 (81.1%) 25 (18.9%)	30 (65.2%) 13 (28.3%) 3 (6.5%) 73 (79.3%) 19 (20.7%)	I − III: 4.0, 0.14 **I − II + III: 6.175, 0.046** I + II − III: 0.74, 0.69 I − III: 3.49, 0.062 **I − II + III: 6.09, 0.014** I + II − III: 0.47, 0.49
rs4426765 AA AC CC A C	25 (64.1%) 8 (20.5%) 6 (15.4%) 58 (74.4%) 20 (25.6%)	33 (50.0%) 29 (43.9%) 4 (6.1%) 95 (72.0%) 37 (28.0%)	29 (63.0%) 16 (34.8%) 1 (2.2%) 74 (80.4%) 18 (19.6%)	**I − III: 6.0, 0.05** **I − II + III: 8.31, 0.016** I + II − III: 2.71, 0.26 I − III: 0.9, 0.34 I − II + III: 0.037, 0.85 I + II − III: 1.97, 0.16
rs28649799 AA AG GG A G	30 (76.9%) 9 (23.1%) 0 69 (88.5%) 9 (11.5%)	58 (87.9%) 7 (10.6%) 1 (1.5%) 123 (93.2%) 9 (6.8%)	35 (76.1%) 9 (19.6%) 2 (4.3%) 79 (85.9%) 13 (14.1%)	I − III: 1.82, 0.4 I − II + III: 2.53, 0.28 I + II − III: 2.45, 0.29 I − III: 0.25, 0.62 I − II + III: 0.19, 0.67 I + II − III: 2.15, 0.14
rs4364254 TT TC CC T C	20 (51.3%) 15 (38.5%) 4 (10.3%) 55 (70.5%) 23 (29.5%)	33 (50.0%) 26 (37.9%) 8 (12.1%) 91 (68.9%) 41 (31.1%)	23 (50.0%) 18 (39.1%) 5 (10.9%) 64 (69.6%) 28 (30.4%)	I − III: 0.017, 0.99 I − II + III: 0.059, 0.97 I + II − III: 0.008, 0.99 I − III: 0.018, 0.89 I − II + III: 0.047, 0.83 I + II − III: 0, 1

Significant deviations (*p* < 0.05) are marked in bold.

**Table 3:** Genotype and allele frequencies of the HPSE gene SNPs in correlation with primary extramedullary disease (EMD) in multiple myeloma.

SNPs/ Genotypes and Alleles	Total EMD			Paraskeletal Disease		
	Yes Number/ Incidence	No Number/ Incidence	χ^2^/ *p*-Value	Yes Number/ Incidence	No Number/ Incidence	χ^2^/ *p*-Value
rs4693608						
AA	9 (18.0%)	59 (36.4%)	**11.98,**	8 (18.6%)	59 (36.4%)	**8.07,**
AG	25 (50.0%)	82 (50.6%)	**0.003**	23 (53.5%)	82 (50.6%)	**0.018**
GG	16 (32.0%)	21 (13%)		12 (27.9%)	21 (13.0%)	
						
A	43 (43.0%)	200 (61.7%)	**10.96,**	39 (45.3%)	200 (61.7%)	**7.5,**
G	57 (57.0%)	124 (38.3%)	**0.001**	47 (54.7%)	124 (38.3%)	**0.006**
rs4693084						
GG	25 (50.0%)	107 (66.5%)	**8.65,**	22 (51.2%)	107 (66.5%)	**5.76,**
GT	18 (36.0%)	48 (29.8%)	**0.013**	16 (37.2%)	48 (29.8%)	**0.056**
TT	7 (14.0%)	6 (3.7%)		5 (11.6%)	6 (3.7%)	
						
G	68 (68.0%)	262 (81.1%)	**7.996,**	60 (69.8%)	262 (81.1%)	**5.49,**
T	32 (32.0%)	60 (18.6%)	**0.005**	26 (30.2%)	60 (18.6%)	**0.019**
rs4426765						
AA	23 (46.0%)	103 (63.6%)	4.94,	18 (41.9%)	103 (63.6%)	**6.77,**
AC	22 (44.0%)	49 (30.2%)	0.085	20 (46.5%)	49 (30.2%)	**0.034**
CC	5 (10.0%)	10 (6.2%)		5 (11.6%)	10 (6.2%)	
						
A	68 (68.0%)	255 (78.7%)	**4.82,**	56 (65.1%)	255 (78.7%)	**6.85,**
C	32 (32.0%)	69 (21.3%)	**0.028**	30 (34.9%)	69 (21.3%)	**0.009**
rs28649799						
AA	35 (70.0%)	135 (83.3%)	4.33,	32 (74.4%)	135 (83.3%)	3.44,
AG	13 (26.0%)	24 (14.8%)	0.12	11 (25.6%)	24 (14.8%)	0.18
GG	2 (4.0%)	3 (1.9%)		0	3 (1.9%)	
						
A	83 (83.0%)	294 (90.7%)	**4.65,**	75 (87.2%)	294 (90.7%)	0.94,
G	17 (17.0%)	30 (9.3%)	**0.031**	11 (12.8%)	30 (9.3%)	0.33
rs4364254						
TT	17 (34.0%)	97 (59.9%)	**11.42,**	16 (37.2%)	97 (59.9%)	**7.92,**
TC	23 (46.0%)	51 (31.5%)	**0.003**	19 (44.2%)	51 (31.5%)	**0.019**
CC	10 (20.0%)	14 (8.6%)		8 (18.6%)	14 (8.6%)	
						
T	57 (57.0%)	245 (75.6%)	**12.92,**	51 (59.3%)	245 (75.6%)	**9.01,**
C	43 (43.0%)	79 (24.4%)	**0.00032**	35 (40.7%)	79 (24.4%)	**0.003**
rs4693608						
rs4364254						
HR	9 (18.0%)	59 (36.4%)	**17.99,**	8 (18.6%)	59 (36.4%)	**14.32,**
MR	19 (38.0%)	77 (47.5%)	**0.00012**	17 (39.5%)	77 (47.5%)	**0.00078**
LR	22 (44.0%)	26 (16.1%)	**Yc: 16.036, 0.00033**	18 (41.9%)	26 (16.1%)	**Yc: 12.472, 0.002**

Significant deviations (*p* < 0.05) are marked in bold. Yc—Yates’ correction.

**Table 4:** Genotype and allele frequencies of the HPSE2 gene SNPs in correlation with primary extramedullary disease (EMD) in multiple myeloma.

SNPs/ Genotypes and Alleles	Total EMD			Paraskeletal Disease		
	Yes Number/ Incidence	No Number/ Incidence	χ^2^/ *p*-Value	Yes Number/ Incidence	No Number/ Incidence	χ^2^/ *p*-Value
rs2040022						
AA	19 (38%)	57 (35.2%)	NS	15 (34.9%)	57 (35.2%)	NS
AG	21 (42%)	81 (50.0%)		19 (44.2%)	81 (50.0%)	
GG	10 (20%)	24 (14.8%)		9 (20.9%)	24 (14.8%)	
						
A	59 (59%)	195 (60.2%)	NS	49 (57.0%)	195 (60.2%)	NS
G	41 (41%)	129 (39.8%)		37 (43.0%)	129 (39.8%)	
rs658225						
AA	32 (64%)	110 (67.9%)	NS	26 (60.5%)	110 (67.9%)	NS
AG	15 (30%)	50 (30.9%)		15 (34.9%)	50 (30.9%)	
GG	3 (6%)	2 (1.2%)		2 (4.7%)	2 (1.2%)	
						
A	79 (79%)	270 (83.3%)	NS	67 (77.9%)	270 (83.3%)	NS
G	21 (21%)	54 (16.7%)		19 (22.1%)	54 (6.7%)	
rs7081116						
AA	30 (60%)	99 (61.9%)	NS	25 (58.1%)	99 (61.9%)	NS
AG	17 (34%)	55 (34.4%)		15 (34.9%)	55 (34.4%)	
GG	3 (6%)	6 (3.8%)		3 (7.0%)	6 (3.8%)	
						
A	77 (77%)	253 (79.1%)	NS	65 (75.6%)	253 (79.1%)	NS
G	23 (23%)	67 (20.9%)		21 (24.4%)	67 (20.9%)	
rs861399						
TT	36 (72%)	111 (68.5%)	NS	31 (72.1%)	111 (68.5%)	NS
TC	13 (26%)	49 (30.2%)		11 (25.6%)	49 (30.2%)	
CC	2 (1%)	2 (1.2%)		1 (2.3%)	2 (1.2%)	
						
T	85 (85%)	271 (83.6%)	NS	73 (84.9%)	271 (83.6%)	NS
C	15 (15%)	53 (16.4%)		13 (15.1%)	53 (16.4%)	
rs7076702						
CC	29 (58%)	95 (58.6%)	NS	24 (55.8%)	95 (58.6%)	NS
CG	18 (36%)	59 (36.4%)		16 (37.2%)	59 (36.4%)	
GG	3 (6%)	8 (4.9%)		3 (7.0%)	8 (4.9%)	
						
C	76 (76%)	249 (76.9%)	NS	64 (74.4%)	249 (76.9%)	NS
G	24 (24%)	75 (23.1%)		22 (25.6%)	75 (23.1%)	
rs475711						
CC	28 (56%)	101 (62.3%)	NS	23 (53.5%)	101 (62.3%)	NS
CT	19 (38%)	56 (34.6%)		17 (39.5%)	56 (34.6%)	
TT	3 (6%)	5 (3.1%)		3 (7.0%)	5 (3.1%)	
						
C	75 (75%)	258 (79.6%)	NS	63 (73.3%)	258 (79.6%)	NS
T	25 (25%)	66 (20.4%)		23 (26.7%)	66 (20.4%)	
rs657442						
CC	50 (100%)	140 (86.4%)	**0.0065**	43 (100%)	140 (86.4%)	**0.011**
CA	0	21 (13.0%)		0	21 (13.0%)	
AA	0	1 (0.6%)		0	1 (0.6%)	
						
C	100 (100%)	301 (92.9%)	**0.0038**	86 (100%)	301 (92.9%)	**0.0066**
A	0	23 (7.1%)		0	23 (7.1%)	
rs57455933						
AA	26 (52%)	75 (46.3%)	NS	23 (53.5%)	75 (46.3%)	NS
AT	21 (42%)	68 (42.0%)		18 (41.7%)	68 (42.0%)	
TT	3 (6%)	19 (11.7%)		2 (4.7%)	19 (11.7%)	
						
A	73 (73%)	218 (67.3%)	NS	64 (74.4%)	218 (67.3%)	NS
T	27 (27%)	106 (32.7%)		22 (25.6%)	106 (32.7%)	
rs621644						
GG	25 (50%)	85 (52.8%)	NS	22 (51.2%)	85 (52.8%)	NS
GA	22 (44%)	65 (40.4%)		19 (44.2%)	65 (40.4%)	
AA	3 (6%)	11 (6.8%)		2 (4.7%)	11 (6.8%)	
						
G	72 (72%)	235 (73%)	NS	63 (73.3%)	235 (73%)	NS
A	28 (28%)	87 (27%)		23 (26.7%)	87 (27%)	
rs1998756						
CC	20 (40%)	74 (45.7%)	NS	18 (41.9%)	74 (45.7%)	NS
CT	24 (48%)	71 (43.8%)		20 (46.5%)	71 (43.8%)	
TT	6 (12%)	17 (10.5%)		5 (11.6%)	17 (10.5%)	
						
C	64 (64%)	219 (67.6%)	NS	56 (65.1%)	219 (67.6%)	NS
T	36 (36%)	105 (32.4%)		30 (34.9%)	105 (32.4%)	
rs754585						
CC	10 (20%)	39 (24.1%)	NS	8 (18.6%)	39 (24.1%)	NS
CT	29 (58%)	77 (47.5%)		27 (62.8%)	77 (47.5%)	
TT	11 (22%)	46 (28.4%)		8 (18.6%)	46 (28.4%)	
						
C	49 (49%)	155 (47.8%)	NS	43 (50.0%)	155 (47.8%)	NS
T	51 (51%)	169 (52.2%)		43 (50.0%)	169 (52.2%)	

Significant deviations (*p* < 0.05) are marked in bold.

**Table 5:** Examination of the interaction between HPSE rs4693608 and rs4364254 SNPs and HPSE2 rs657422 SNP in correlation with primary extramedullary disease (EMD) of multiple myeloma.

Genotypes	Yes Number/Incidence	No Number/Incidence	χ^2^/ *p*-Value
HR-CC	9 (18.0%)	50 (30.9%)	
HR-CA	0	8 (4.9%)	
HR-AA	0	1 (0.6%)	
		
MR-CC	19 (38.0%)	69 (42.6%)	
MR-CA	0	8 (4.9%)	
MR-AA	0	0	
		
LR-CC	22 (44.0%)	21 (13.0)	
LR-CA	0	5 (3.1%)	
LR-AA	0	0	
Group A1 Group A2 Group A3	9 (18.0%) 19 (38.0%) 22 (44.0%)	67 (41.4%) 74 (45.7%) 21 (12.9%)	**χ^2^_df2_ = 22.47,** ***p* = 0.0000049** **Yates’ χ^2^ = 22.18,** **Yates’ *p* = 0.000015**
Group B1 Group B2 Group B3	9 (18.0%) 19 (38.0%) 22 (44.0%)	72 (44.4%) 69 (42.6%) 21 (13.0%)	**χ^2^_df2_ = 25.33,** ***p* = 0.0000036** **Yates’ χ^2^ = 23.065,** **Yates’ *p* = 0.0000098**

Significant deviations (*p* < 0.05) are marked in bold. Group A1: HR-CC, HR-CA, HR-AA, MR-CA; group A2: MR-CC, LR-CA; group A3: LR-CC. Group B1: HR-CC, HR-CA, HR-AA, MR-CA, LR-CA; group B2: MR-CC; group B3: LR-CC.

**Table 6:** Genotype and allele frequencies of the HPSE2 gene SNPs in correlation with secondary extramedullary disease (EMD) in multiple myeloma.

SNPs/ Genotypes and Alleles	Total EMD			Paraskeletal Disease		
	Yes Number/ Incidence	No Number/ Incidence	χ^2^/ *p*-Value	Yes Number/ Incidence	No Number/ Incidence	χ^2^/ *p*-Value
rs2040022						
AA	15 (40.5%)	41 (34.2%)	NS	9 (32.1%)	41 (34.2%)	NS
AG	16 (43.2%)	61 (50.8%)		14 (50.0%)	61 (50.8%)	
GG	6 (16.2%)	18 (15.0%)		5 (17.9%)	18 (15.0%)	
						
A	46 (62.1%)	143 (59.6%)	NS	32 (57.1%)	143 (59.6%)	NS
G	28 (37.8%)	97 (40.4%)		24 (42.9%)	97 (40.4%)	
rs658225						
AA	31 (83.8%)	77 (64.2%)	5.24,	23 (82.1%)	77 (64.2%)	3.5,
AG	6 (16.2%)	41 (34.2%)	0.073	5 (17.9%)	41 (34.2%)	0.17
GG	0	2 (1.7%)		0	2 (1.7%)	
						
A	68 (91.9%)	195 (81.3%)	**4.71,**	51 (91.1%)	195 (81.3%)	3.12,
G	6 (8.1%)	45 (18.7%)	**0.03**	5 (8.9%)	45 (18.7%)	0.077
rs7081116						
AA	24 (64.9%)	72 (61.0%)	NS	16 (57.1%)	72 (61.0%)	NS
AG	11 (29.7%)	42 (35.6%)		11 (39.3%)	42 (35.6%)	
GG	2 (5.4%)	4 (3.4%)		1 (3.6%)	4 (3.4%)	
						
A	59 (79.7%)	186 (78.8%)	NS	43 (76.8%)	186 (78.8%)	NS
G	15 (20.3%)	50 (21.2%)		13 (23.2%)	50 (21.2%)	
rs861399						
TT	25 (67.6%)	82 (68.3%)	NS	17 (60.7%)	82 (68.3%)	NS
TC	11 (29.7%)	37 (30.8%)		11 (39.3%)	37 (30.8%)	
CC	1 (2.7%)	1 (0.8%)		0	1 (0.8%)	
						
T	61 (82.4%)	201 (83.7%)	NS	45 (80.4%)	201 (83.7%)	NS
C	13 (17.6%)	39 (16.3%)		11 (19.6%)	39 (16.3%)	
rs7076702						
CC	24 (64.9%)	67 (55.8%)	NS	16 (57.1%)	67 (55.8%)	NS
CG	11 (29.7%)	47 (39.2%)		11 (39.3%)	47 (39.2%)	
GG	2 (5.4%)	6 (5.0%)		1 (3.6%)	6 (5.0%)	
						
C	59 (79.7%)	181 (75.4%)	NS	43 (76.8%)	181 (75.4%)	NS
G	15 (20.3%)	59 (24.6%)		13 (23.2%)	59 (24.6%)	
rs475711						
CC	23 (62.2%)	75 (62.5%)	NS	16 (57.1%)	75 (62.5%)	NS
CT	13 (35.1%)	41 (34.2%)		12 (42.9%)	41 (34.2%)	
TT	1 (2.7%)	4 (3.3%)		0	4 (3.3%)	
						
C	59 (79.7%)	191 (79.6%)	NS	44 (78.6%)	191 (79.6%)	NS
T	15 (20.3%)	49 (20.4%)		12 (21.4%)	49 (20.4%)	
rs657442						
CC	34 (91.9%)	102 (85.0%)	NS	26 (92.9%)	102 (85.0%)	NS
CA	3 (8.1%)	17 (14.2%)		2 (7.1%)	17 (14.2%)	
AA	0	1 (0.8%)		0	1 (0.8%)	
						
C	71 (95.9%)	221 (92.1%)	NS	54 (96.4%)	221 (92.1%)	NS
A	3 (4.1%)	19 (7.9%)		2 (3.6%)	19 (7.9%)	
rs57455933						
AA	20 (54.1%)	53 (44.2%)	NS	14 (50.0%)	53 (44.2%)	NS
AT	11 (29.7%)	54 (45.0%)		8 (28.6%)	54 (45.0%)	
TT	6 (16.2%)	13 (10.8%)		6 (21.4%)	13 (10.8%)	
						
A	51 (68.9%)	160 (66.7%)	NS	36 (64.3%)	160 (66.7%)	NS
T	23 (31.1%)	106 (33.3%)		20 (35.7%)	106 (33.3%)	
rs621644						
GG	20 (54.1%)	62 (52.1%)	**7.16,**	13 (46.4%)	62 (52.1%)	**9.9,**
GA	11 (29.7%)	52 (43.7%)	**0.028**	9 (32.1%)	52 (43.7%)	**0.007**
AA	6 (16.2%)	5 (4.2%)		6 (21.4%)	5 (4.2%)	
						
G	51 (68.9%)	176 (73.9%)	NS	35 (62.5%)	176 (73.9%)	2.93,
A	23 (31.1%)	62 (26.1%)		21 (37.5%)	62 (26.1%)	0.087
rs1998756						
CC	18 (48.6%)	54 (45.0%)	NS	14 (50.0%)	54 (45.0%)	NS
CT	15 (40.5%)	54 (45.0%)		11 (39.3%)	54 (45.0%)	
TT	4 (10.8%)	12 (10.0%)		3 (10.7%)	12 (10.0%)	
						
C	51 (68.9%)	162 (67.5%)	NS	39 (69.6%)	162 (67.5%)	NS
T	23 (31.1%)	78 (32.5%)		17 (30.4%)	78 (32.5%)	
rs754585						
CC	8 (21.6%)	30 (25.0%)	**7.24,**	6 (21.4%)	30 (25.0%)	5.42,
CT	24 (64.9%)	50 (41.7%)	**0.027**	18 (64.3%)	50 (41.7%)	0.067
TT	5 (13.5%)	40 (33.3%)		4 (14.3%)	40 (33.3%)	
						
C	40 (54.1%)	155 (45.8%)	NS	30 (53.6%)	155 (45.8%)	NS
T	34 (45.9%)	169 (54.2%)		26 (46.4%)	169 (54.2%)	

Significant deviations (*p* < 0.05) are marked in bold.

**Table 7:** Examination of the interaction between HPSE2 gene SNPs rs658225 and rs754585 in correlation with secondary extramedullary disease (EMD) of multiple myeloma.

Genotypes	Secondary EMD Number/Incidence	Total EMD Number/Incidence	No Number/Incidence
AA-CC	8 (21.1%)	13 (14.9%)	22 (18.3%)
AA-CT	**20 (52.6%)**	**40 (46.0%)**	**28 (23.3%)**
AA-TT	**4 (10.5%)**	**10 (11.5%)**	**27 (22.5%)**
			
AG-CC	1 (2.6%)	4 (4.6%)	8 (6.7%)
AG-CT	4 (10.5%)	12 (13.8%)	22 (18.3%)
AG-TT	1 (2.6%)	5 (5.8%)	11 (9.2%)
			
GG-CC	0	1 (1.1)	0
GG-CT	0	1 (1.1)	0
GG-TT	0	1 (1.1)	2 (1.7%)

Significant deviations are marked in bold.

**Table 8:** Genotype and allele frequencies of the HPSE2 gene SNPs in correlation with bone morbidity in multiple myeloma patients.

SNPs	Genotypes and Alleles	Yes Number/Incidence	No Number/Incidence	χ^2^/ *p*-Value
rs2040022	AA	56 (35.4%)	17 (32.1%)	NS NS
	AG	78 (49.4%)	26 (49.0%)	
	GG	24 (15.2%)	10 (18.9%)	
		
	A	190 (60.1%)	60 (56.6%)	
	G	126 (39.9%)	46 (43.4%)	
rs658225	AA	109 (69.0%)	34 (64.2%)	NS NS
	AG	44 (27.8%)	19 (35.8%)	
	GG	5 (3.2%)	0	
		
	A	262 (82.9%)	87 (82.1%)	
	G	54 (17.1%)	19 (17.9%)	
rs7081116	AA	91 (58.3%)	35 (66.0%)	NS NS
	AG	56 (35.9%)	18 (34.0%)	
	GG	9 (5.8%)	0	
		
	A	238 (76.3%)	88 (83.0%)	
	G	74 (23.7%)	18 (17.0%)	
rs861399	TT	104 (65.8%)	40 (75.5%)	NS NS
	TC	51 (32.3%)	13 (24.5%)	
	CC	3 (1.9%)	0	
		
	T	259 (82.0%)	93 (87.3%)	
	C	57 (18.0%)	13 (12.3%)	
rs7076702	CC	87 (55.1%)	34 (64.2%)	NS NS
	CG	61 (38.6%)	18 (34.0%)	
	GG	10 (6.3%)	1 (1.9%)	
		
	C	235 (74.4%)	86 (81.1%)	
	G	81 (25.6%)	20 (18.9%)	
rs475711	CC	90 (57.0%)	36 (67.9%)	NS NS
	CT	60 (38.0%)	17 (32.1%)	
	TT	8 (5.0%)	0	
		
	C	240 (76.0%)	89 (84.0%)	
	T	76 (24.0%)	17 (16.0%)	
rs657442	CC	149 (94.3%)	41 (77.4%)	**13.74,**
	CA	9 (5.7%)	11 (20.8%)	**0.001**
	AA	0	1 (1.9%)	
			
	C	307 (97.2%)	93 (87.7%)	**14.24,**
	A	9 (2.8%)	13 (12.3%)	**0.00016**
rs57455933	AA	77 (48.7%)	24 (45.3%)	
	AT	67 (42.4%)	20 (37.7%)	NS
	TT	14 (8.9%)	9 (17.0%)	
			
	A	221 (69.9%)	68 (64.2%)	
	T	95 (30.1%)	38 (35.4%)	NS
rs621644	GG	81 (51.7%)	28 (52.8%)	
	GA	64 (40.8%)	22 (41.5%)	NS
	AA	12 (7.6%)	3 (5.7%)	
			
	G	226 (72.0%)	78 (73.6%)	
	A	88 (28.0%)	28 (26.4%)	NS
rs1998756	CC	74 (46.8%)	20 (37.7%)	
	CT	66 (41.8%)	27 (51.0%)	NS
	TT	18 (11.4%)	6 (11.3%)	
			
	C	214 (67.7%)	67 (63.2%)	
	T	102 (32.3%)	39 (36.8%)	NS
rs754585	CC	37 (23.4%)	13 (24.5%)	
	CT	80 (50.6%)	25 (47.2%)	NS
	TT	41 (25.9%)	15 (28.3%)	
			
	C	154 (48.7%)	51 (48.1%)	
	T	162 (51.3%)	55 (51.9%)	NS

Significant deviations (*p* < 0.05) are marked in bold.

## Data Availability

Not applicable.

## Supplementary Materials

The following supporting information can be downloaded at: https://www.mdpi.com/article/10.3390/cells12060913/s1, Table S1: Genotype and allele frequencies of the HPSE gene SNPs in multiple myeloma patients.
