# Supplementary material for: Effect of HPSE and HPSE2 SNPs on the Risk of Developing Primary Paraskeletal Multiple Myeloma
Source: Cells. 2023 Mar 16;12(6):913. doi: 10.3390/cells12060913 (PMC10047783; doi:10.3390/cells12060913)
Supplement: Supplementary file 1 [file cells-12-00913-s001.zip › cells-2199480-supplementary.pdf]

Supplementary Table S1. Genotype and allele frequencies of the HPSE gene SNPs in multiple myeloma patients.

SNPs	Genotypes and alleles	Total MM		Active MM		Controls
		Number/ incidence	χ^2/ p-value	Number / incidence	χ^2/ p-value	Number / incidence
rs4693608	AA	87 (31.8%)	NS	64 (28.2%)	NS	58 (27.5%)
	AG	140 (51.1%)		123 (54.2%)		112 (53.1%)
	GG	47 (17.2%)		40 (17.6%)		41 (19.4%)
	A	314 (57.3%)	NS	251 (55.3%)	NS	228 (54.0%)
	G	234 (42.7%)		203 (44.7%)		194 (46.0%)
rs4693084	GG	169 (62.6%)	NS	135 (60.5%)	NS	131 (62.1%)
	GT	87 (32.2%)		74 (33.2%)		71 (33.6%)
	TT	14 (5.2 %)		14 (6.3%)		9 (4.3%)
	G	425 (78.7%)	NS	344 (77.1%)	NS	333 (78.9%)
	T	115 (21.3%)		102 (22.9%)		89 (21.1%)
rs4426765	AA	159 (58.5%)	NS	133 (59.1%)	NS	120 (56.9%)
	AC	92 (33.8%)		76 (33.8%)		71 (33.6%)
	CC	21 (7.7%)		16 (7.1%)		20 (9.5%)
	A	410 (75.4%)	NS	342 (76.0%)	NS	311 (73.7%)
	C	134 (24.6%)		108 (24.0%)		111 (26.3%)
rs28649799	AA	225 (82.7%)	NS	181 (80.4%)	NS	169 (80.1%)
	AG	42 (15.4%)		39 (17.3%)		41 (19.4%)
	GG	5 (1.8%)		5 (2.2%)		1 (0.5%)
			NS		NS	
	A	492 (90.4%)		401 (89.1%)		379 (89.8%)
	G	52 (9.6%)		49 (10.9%)		43 (10.2%)
rs4364254	TT	150 (54.7%)	**5.04,**	123 (54.2%)	**4.4,**	96 (45.5%)
	TC	95 (34.7%)	**0.08**	79 (34.8%)	**0.11**	94 (44.5%)
	CC	29 (10.6%)		25 (11.0%)		21 (10.0%)
	T	395 (72.1%)	NS	325 (71.6%)	NS	286 (67.8%)
	C	153 (27.9%)		129 (28.4%)		136 (32.2%)

Significant deviations ($p<0.05$) are marked in bold.

Total MM group is included patients with multiple myeloma (MM), monoclonal gammopathy (MGUS) and smoldering multiple myeloma (SMM) diseases.
